# Antimalarial antibody repertoire defined by plasma IG proteomics and single B cell IG sequencing

**DOI:** 10.1172/jci.insight.143471

**Published:** 2020-11-19

**Authors:** Camila H. Coelho, Steven T. Nadakal, Patricia Gonzales Hurtado, Robert Morrison, Jacob D. Galson, Jillian Neal, Yimin Wu, C. Richter King, Virginia Price, Kazutoyo Miura, Sharon Wong-Madden, Justin Yai Alamou Doritchamou, David L. Narum, Nicholas J. MacDonald, Maryonne Snow-Smith, Marissa Vignali, Justin J. Taylor, Marie-Paule Lefranc, Johannes Trück, Carole A. Long, Issaka Sagara, Michal Fried, Patrick E. Duffy

**Affiliations:** 1 Laboratory of Malaria Immunology and Vaccinology, National Institute of Allergy and Infectious Diseases, NIH, Bethesda, Maryland, USA.; 2 University Children’s Hospital Zurich, Zurich, Switzerland.; 3 Alchemab Therapeutics Ltd, London, United Kingdom.; 4 PATH’s Malaria Vaccine Initiative, Washington, DC, USA.; 5 Laboratory of Malaria and Vector and Research, National Institute of Allergy and Infectious Diseases, NIH, Rockville, Maryland, USA.; 6 Adaptive Biotechnologies Corp, Seattle, Washington, USA.; 7 Fred Hutchinson Cancer Research Center, Seattle, Washington, USA.; 8 IMGT, the International ImMunoGeneTics Information System, Laboratoire d’ImmunoGénétique Moléculaire, Institut de Génétique Humaine, UMR9002 CNRS, Université de Montpellier, Montpellier, France.; 9 Malaria Research and Training Center, University of Sciences, Techniques, and Technologies, Bamako, Mali.

**Keywords:** Immunology, Vaccines, Adaptive immunity, Malaria

## Abstract

Plasma antimalarial Ab can mediate antiparasite immunity but has not previously been characterized at the molecular level. Here, we develop an innovative strategy to characterize humoral responses by integrating profiles of plasma immunoglobulins (IGs) or Abs with those expressed on B cells as part of the B cell receptor. We applied this strategy to define plasma IG and to determine variable (V) gene usage after vaccination with the *Plasmodium falciparum* zygote antigen Pfs25. Using proteomic tools coupled with bulk immunosequencing data, we determined human antigen-binding fragment [F(ab′)_2_] peptide sequences from plasma IG of adults who received 4 doses of Pfs25-EPA/Alhydrogel. Specifically, Pfs25 antigen-specific F(ab′)_2_ peptides (Pfs25-IG) were aligned to cDNA sequences of IG heavy (IGH) chain complementarity determining region 3 from a data set generated by total peripheral B cell immunosequencing of the entire vaccinated population. IGHV4 was the most commonly identified IGHV subgroup of Pfs25-IG, a pattern that was corroborated by V heavy/V light chain sequencing of Pfs25-specific single B cells from 5 vaccinees and by matching plasma Pfs25-IG peptides and V-(D)-J sequences of Pfs25-specific single B cells from the same donor. Among 13 recombinant human mAbs generated from IG sequences of Pfs25-specific single B cells, a single IGHV4 mAb displayed strong neutralizing activity, reducing the number of *P*. *falciparum* oocysts in infected mosquitoes by more than 80% at 100 μg/mL. Our approach characterizes the human plasma Ab repertoire in response to the Pfs25-EPA/Alhydrogel vaccine and will be useful for studying circulating Abs in response to other vaccines as well as those induced during infections or autoimmune disorders.

## Abstract 2

An approach to characterize the human plasma antibody repertoire is applied to define plasma Ig and determine variable V gene usage after malaria vaccination.

## Graphical abstract

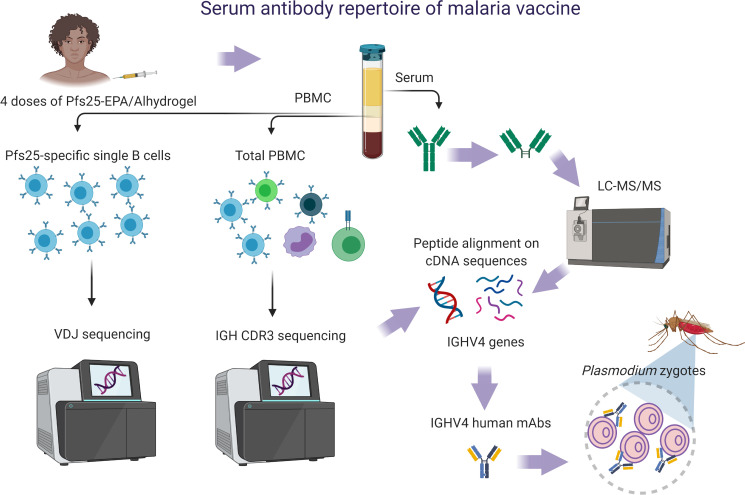

## Introduction

Despite progress on malaria prevention and treatment (1, 2), eradication of this disease will require novel interventions. Transmission blocking vaccines (TBVs) prevent parasite spread through the vector by inducing Abs to surface antigens of mosquito sexual stage development of *Plasmodium falciparum* (3–6). The *P*. *falciparum* zygote/ookinete protein Pfs25 has been the leading TBV candidate antigen for 3 decades and induces Abs that neutralize sexual stage parasites in laboratory assays (7, 8). Pfs25 has advanced to clinical trials in endemic settings but has shown limited potency and variable (V) serum functional activity. The molecular definition of the serum Ab repertoire may explain this limitation and guide the design of improved Pfs25 vaccines. Although numerous rodent studies have analyzed the functional activity of Pfs25 Abs (9–11), detailed characterization of such Abs present in human sera after vaccination has not yet been performed for this or any other malaria vaccine. As such, the identity of Pfs25-specific Abs secreted in sera remains unknown. One approach to identify antigen-specific features of vaccine Ab responses involves the determination of V gene utilization in the B cell receptor (BcR) (12, 13). Convergent V gene responses can be used to design novel immunogens that target specific Ab genes related to protection (14).

Recently, fragments encoding V heavy (VH) and V light (VL) domains obtained from antigen-specific B cells in mice and from plasmablasts of humans immunized with Pfs25 have been sequenced (11, 15). Subsequent studies identified the corresponding Ab epitopes in Pfs25. In that work, immunoglobin HV3 (IGHV3) subgroup sequences from plasmablasts of a single vaccinee with high serum functional activity yielded recombinant Ab that mediated transmission-reducing activity (TRA) (15).

However, Ab repertoire differs between plasma and B cells (16), and plasma Abs convey TRA and, therefore, must be identified and sequenced to characterize the mediators of vaccine activity. In this study, we assessed the plasma Ab repertoire in individuals vaccinated with Pfs25 conjugated to carrier protein Exoprotein A formulated in adjuvant Alhydrogel (Pfs25-EPA/Alhydrogel) during a clinical trial conducted in a high malaria transmission region of Mali (8). We combined proteomic analysis of the antigen-binding fragment F(ab′)_2_ from plasma IG purified on Pfs25 antigen (the plasma proteome data set, referred to herein as plasma Pfs25-IG peptides) with immunosequencing analysis of both the IGH chain complementarity determining region 3 (IGH CDR3) repertoire of total B cells (referred to as IGH CDR3 data set) and the single-cell data set comprising VH/VL of antigen-specific B cells (referred to as Pfs25-specific single B cells).

Here, we report that Abs using the IGHV4 subgroup were the most abundant in the postvaccination plasma proteome and the single-cell data sets. In addition, among the 13 mAbs generated using sequences from the single-cell data set, 2 were functional and both were derived from IGHV4. We demonstrate that peptide sequences of Abs secreted in response to TBV can be used to better characterize Ab-mediated activity. This innovative approach using antigen-specific single B cells as a database to identify V gene sequences of serum IG can be applied to study plasma Ab repertoires in response to other human vaccines or infectious diseases.

## Results

### Vaccination does not significantly alter IGHV gene frequency among total peripheral B cells.

**Figure 1:**
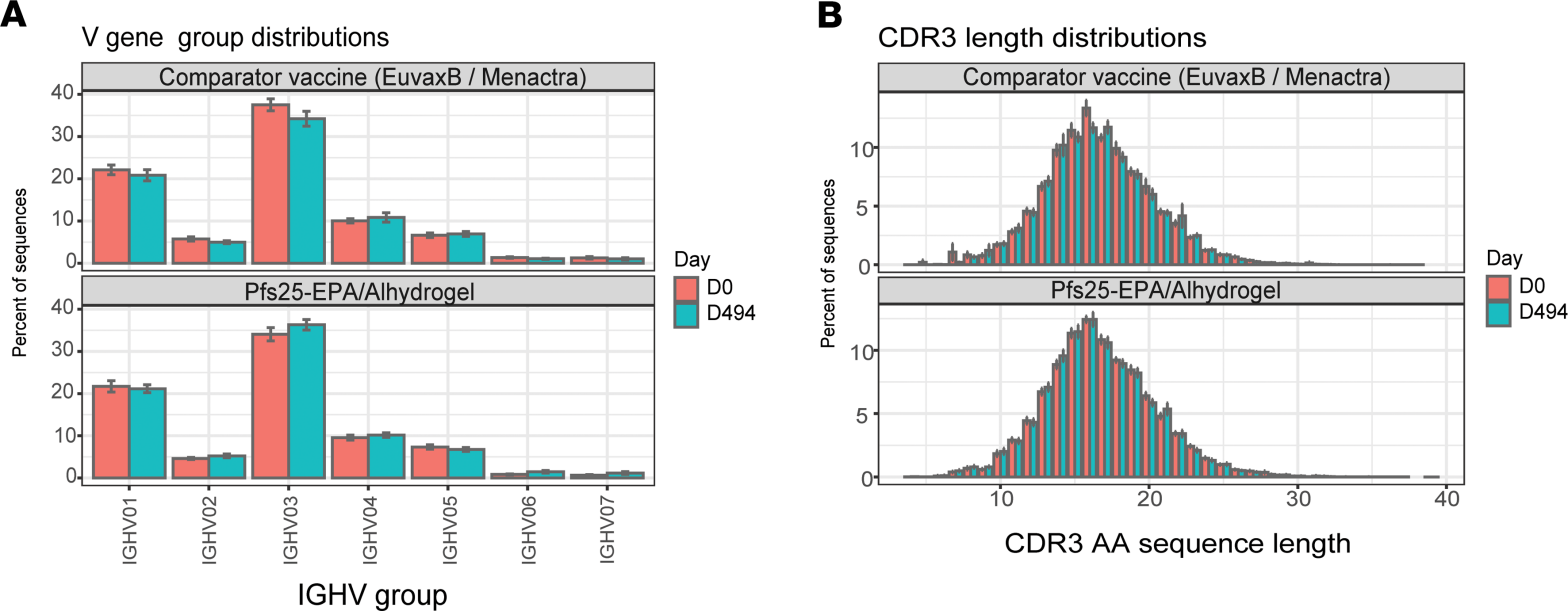
IGHV subgroup usage and IGH CDR3aa lengths for IG of total peripheral B cells before and after Pfs25 or comparator vaccine administration. **(A)** Usage of immunoglobulin heavy (IGHV)subgroups in total peripheral B cells of subjects receiving Pfs25-EPA/Alhydrogel or comparator vaccine (Euvax-B in the first 3 doses and Menactra in dose 4). PBMCs were collected at days 0 and 494 (14 days after dose 4). The complementarity determining region 3 (CDR3) of the IGH chain was amplified by multiplex PCR from cDNA of PBMCs, followed by DNA sequencing to identify and quantitate the absolute abundance of each CDR3. (**B**) Mean CDR3 AA length did not change compared with baseline and did not differ vs. comparator vaccine (*t* test, Supplemental Table 1). Data are shown as mean ± SEM.

The in-house IGH CDR3 data set, generated by the immunoSEQ Assay, comprised 3.16 million sequences obtained 14 days after vaccination from the PBMCs of 79 subjects (vaccinees and comparators) enrolled in the Pfs25-EPA/Alhydrogel clinical trial (mean of 39,966 sequences per subject). Immunosequencing analyses of total B cells after 4 vaccine doses revealed no change in overall IGHV subgroup usage (Supplemental Table 1; supplemental material available online with this article; https://doi.org/10.1172/jci.insight.143471DS1) compared with either baseline or with usage after comparator vaccines (Euvax-B for the first 3 vaccinations and Menactra for dose 4). IGHV3 was the most frequent subgroup in both vaccine and comparator groups at both time points (Figure 1A). Although some other vaccines induce IG with shorter CDR3 length compared with sequences from naive cells (12), no significant differences were identified here in CDR3 length (Figure 1B and Supplemental Table 1) or specific V gene usage (Supplemental Figure 1) between groups and time points.

### Plasma Pfs25-IG peptides detected by mass spectrometry match the international ImMunoGeneTics data set and IGH CDR3 data set.

**Figure 2:**
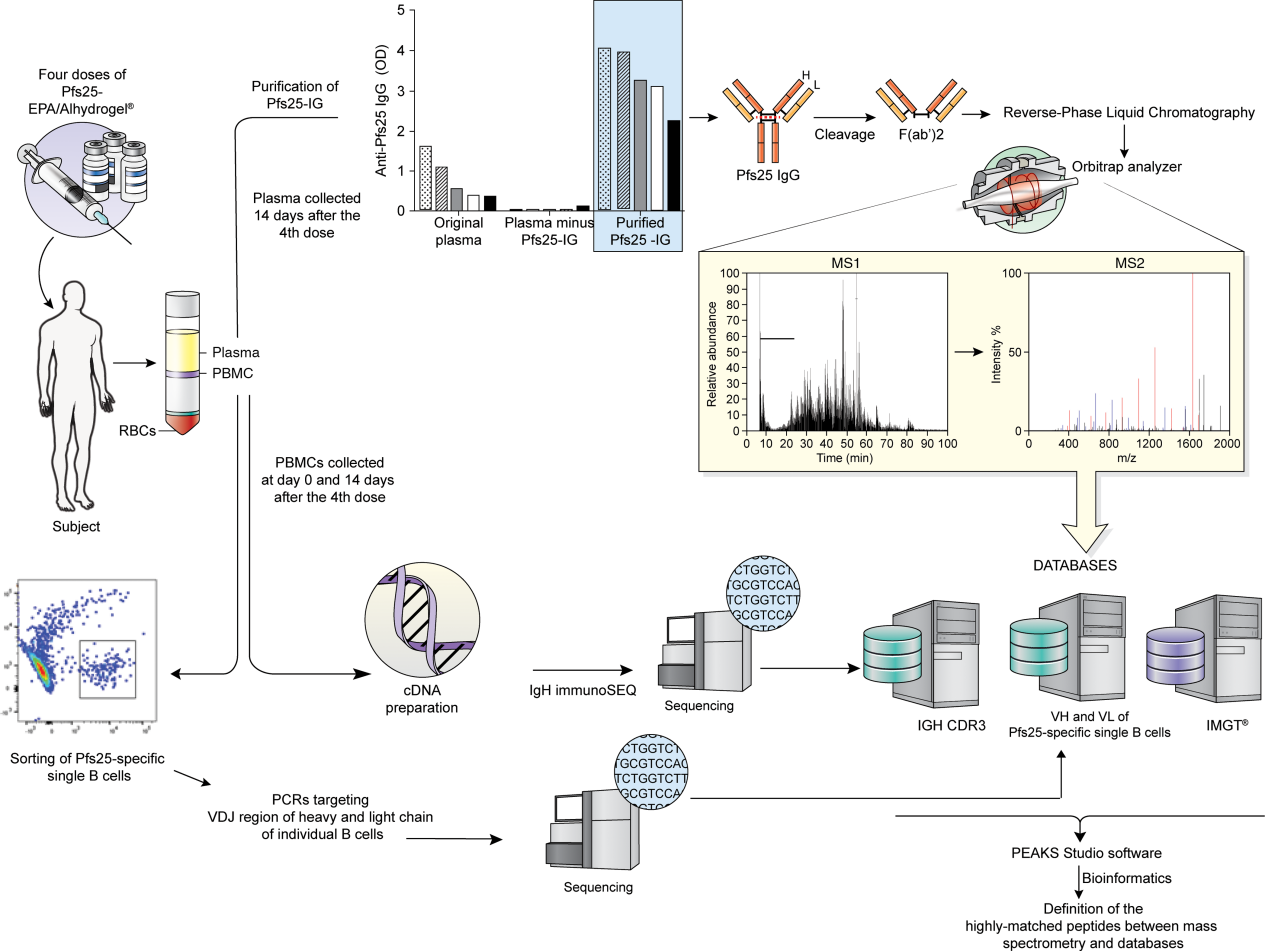
Mass spectrometry analysis pipeline of plasma Pfs25-IG peptides. Plasma and PBMC samples were collected from adults vaccinated with 4 doses of Pfs25 TBV in Mali. Total plasma IG was purified on Pfs25 antigen, and after confirming reactivity to Pfs25 in IgG ELISA, digested and fractionated to isolate the F(ab′)_2_ fragment. Trypsinized F(ab′)_2_ samples were analyzed by Orbitrap MS/MS and the resulting mass spectra were matched to translated sequences from the IMGT germline reference database (17) and those from the in-house IGH CDR3 data set. To match mass spectra to B cell receptor (BcR) sequences of Pfs25-specific single B cells from the same subject (subject 7), the LAX algorithm (18) was used to rank the most frequent matches.

**Figure 3:**
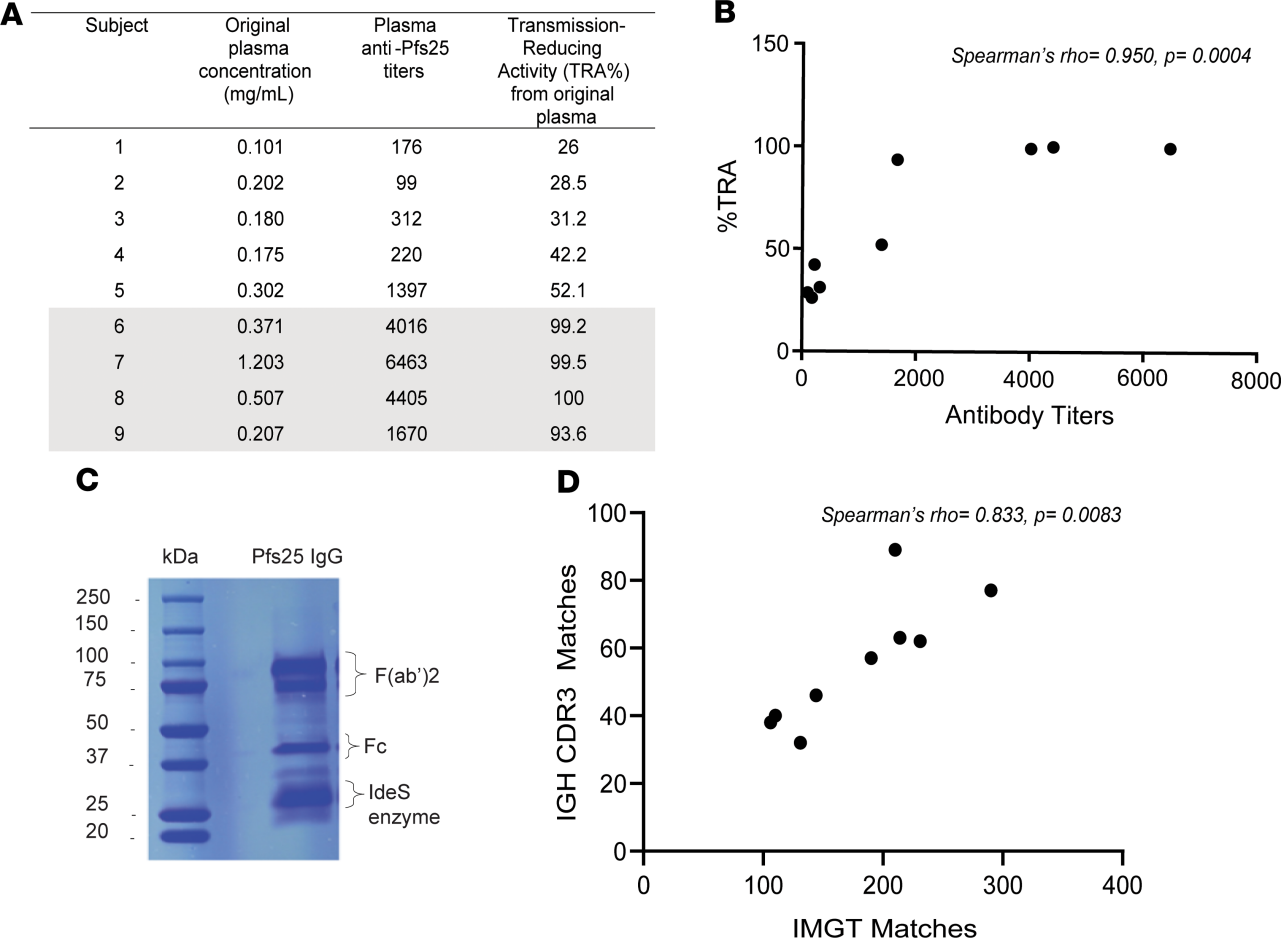
Sample selection for proteomic analysis of serum Pfs25 Abs. (**A**) Serum samples presenting high functional activity (transmission-reducing activity, TRA >90%) are highlighted in gray. Subject ID is listed by increasing level of functional activity, or %TRA. %TRA is calculated as the percentage reduction in the number of oocysts counted on mosquito midguts 8 days after feeding on cultured parasites mixed with individual postvaccination serum and compared with mosquitoes fed with human naive sera. (**B**) Correlation between anti-Pfs25 Ab titers and functional activity (TRA) in sera of vaccinated subjects. (**C**) Coomassie gel containing the digestion products of Pfs25-IG purified from plasma. The region corresponding to F(ab′)_2_ was excised from the gel and analyzed by tandem mass spectrometry (MS/MS). (**D**) Spearman’s correlation between the number of peptide matches in the IMGT database and those in the IGH CDR3 data set was statistically significant.

Pfs25-specific Abs were purified from plasma of 9 Malian adults 14 days after dose 4 of Pfs25-EPA/Alhydrogel vaccine (hereafter referred to as Pfs25-IG) (Figure 2). Original plasma samples were grouped as low or high activity (low activity <55% TRA and <1500 ELISA titer; high activity >90% TRA and >1600 ELISA titer) cohorts based on functional activity and Ab titers (Figure 3A), which correlated significantly (Spearman’s rho = 0.950, *P* = 0.0004) (Figure 3B). By ELISA, Pfs25 titers were high in Pfs25-IG samples and largely absent in the depleted plasma, confirming completeness of Ab purification/depletion (Figure 2 and Supplemental Figure 2). Pfs25-IG from each of the 9 individuals were digested and fractionated separately, then fragments corresponding to the F(ab′)_2_ region were excised from the gel (Figure 3C). Purified Pfs25 F(ab′)_2_ fragments were then analyzed by mass spectrometry and mass spectra were matched to sequences in 2 data sets: the international ImMunoGeneTics (IMGT) and the IGH CDR3 data set (Supplemental Table 2). As expected, matches were more frequent to the public IMGT data set, which contains sequences spanning the entire V region (17), than to the in-house IGH CDR3 data set, which only spans a 43 amino acid (AA) region covering CDR3 (IMGT = 180.6 ± 62.2 peptide matches per subject; IGH CDR3 data set = 56.0 ± 18.9 peptide matches per subject) (Supplemental Figure 3). The number of matches per subject in the 2 databases was highly correlated (Spearman’s rho = 0.833, *P* = 0.0083) (Figure 3D).

### Plasma Pfs25-IG peptides frequently derive from IGHV4 CDR3 sequences.

**Figure 4:**
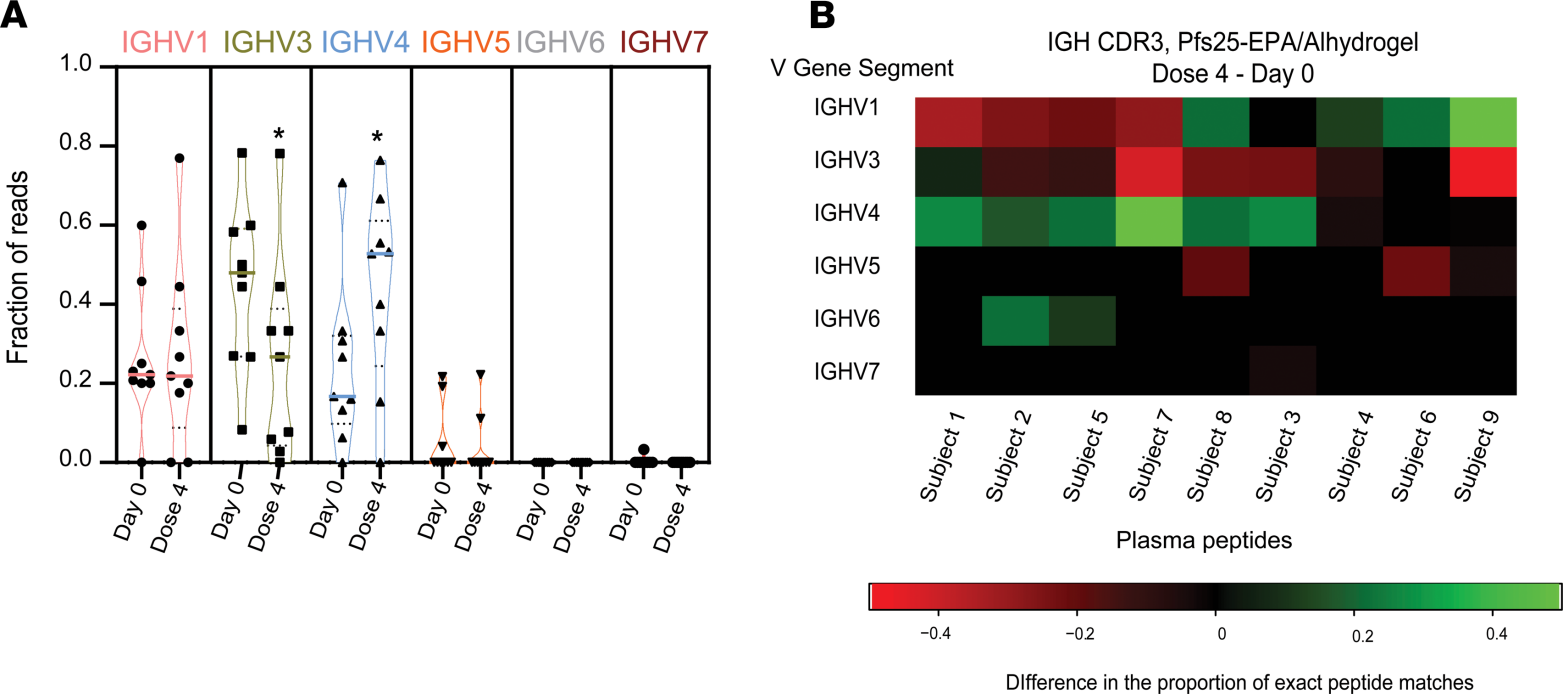
Plasma Pfs25-IG mass spectra matching peptides inferred from sequences in the IGH CDR3 data set before and after Pfs25-EPA/Alhydrogel. **(A)** Proportion of all Pfs25-IG plasma peptides that can be assigned to an IGHV subgroup in the IGH CDR3 data set. Individual dots correspond to the 9 subjects whose plasma samples were used for Pfs25-IG proteomics. Bold colored lines show the median, and black dotted lines show quartiles. Data were calculated using paired *t* test. Raw data are available in Supplemental Table 3. (**B**) Heatmap displays the difference in the proportion of plasma Pfs25-IG peptides that can be attributed to an IGHV subgroup after dose 4 versus day 0 (baseline). Positive values (green) represent a proportional increase in the use of IGHV subgroup after vaccination, and negative values (red) represent a proportional reduction in use. Only IGHV subgroups that yielded matches among the subjects are plotted. Data used to generate the heatmap are shown in Supplemental Table 4, and sequences of the plasma Pfs25-IG peptides identified at baseline and dose 4 matching IGH CDR3 database are provided in Supplemental Table 5.

To assess the effect of vaccination on V gene usage, we compared the alignment of Pfs25-IG mass spectra after vaccination to IGH CDR3 genes at baseline and after vaccination (Supplemental Table 3). Overall, the highest proportion of matches were to IGHV4 based on CDR3 after vaccination, followed by IGHV3 and IGHV1 (Figure 4A and Supplemental Table 4). IGHV4 usage increased after the vaccination compared with the baseline (*P* = 0.0283, paired *t* test) (Figure 4A), whereas usage of IGHV3 in Pfs25-IG plasma peptides decreased after vaccination (*P* = 0.0184, paired *t* test) (Figure 4A). Specifically, 6 of 9 Pfs25 vaccinees showed increase (difference > 0) in the proportion of IGHV4 CDR3 genes mapped to plasma IG peptides after vaccination versus baseline, whereas 8 of 9 vaccinees showed decreases (difference < 0) in IGHV3 (Figure 4B and Supplemental Table 5). Interestingly, the donor presenting the greatest increase in the proportion of IGHV4 usage (subject 7, Figure 4B) had the highest Pfs25 Ab titer (6423 Ab units/mL) and high serum functional activity after vaccination (TRA, 99.5%) (Figure 3A).

### Plasma Pfs25-IG peptides match VH/VL from Pfs25-specific single B cells of the same donor.

**Figure 5:**
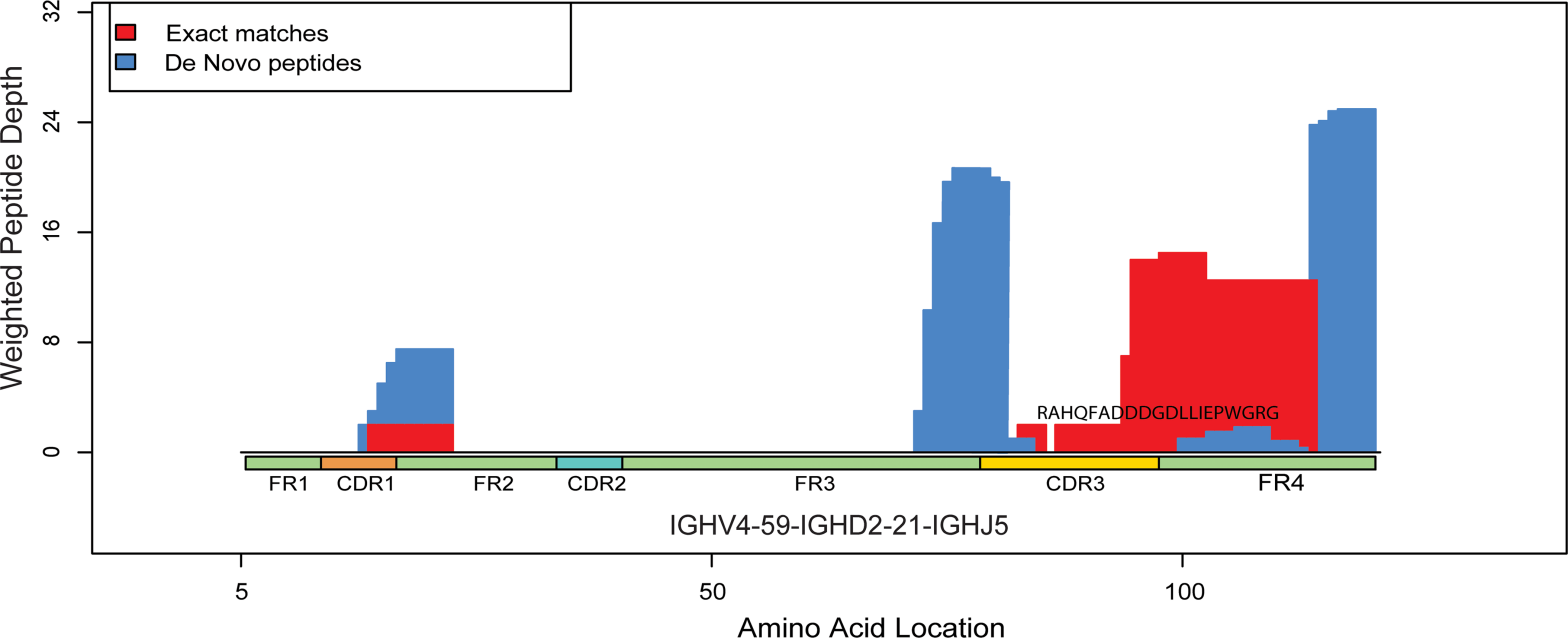
Alignment of IG peptides to VH region from Pfs25-specific B cell of the same donor (subject 7). The figure represents alignment between the most frequently detected plasma IG peptides matching IGHV4 sequence from subject 7. Plasma IG F(ab′)_2_ peptide *RAHQFADDDGDLLIEPWGRG* (20 AA) matched exactly and aligned to a CDR3 sequence of VH obtained from an IGHV4 single B cell in the same donor. Complete information about all plasma peptides from subject 7 matching VH/VL regions of BcR from the same subject are listed in Table 1. Red shows peptides with a perfect match to VH sequence of Pfs25-single B cell from subject 7, whereas blue shows de novo matches (1+ AA mismatches) when aligned to the most similar protein sequence. Peptide weight is calculated by the LAX algorithm to quantify peptide-protein hit uniqueness. Mass spectra that exactly match a peptide from a single protein receive a weight of 1. Mass spectra matching peptides from 2+ proteins equally well have proportionally less weight assigned to the peptide from each protein. Thus, higher weighted peptide depth values along the protein reflect both higher number of peptide alignments and greater peptide uniqueness.

To confirm antigen-specific peptides identified by our approach and to identify peptides shared between single B cells and serum within the same donor, we created a database using VH/VL sequences of Pfs25-specific single B cells (Supplemental Figures 4 and 5) collected from subject 7, who developed the highest Pfs25 titers (Figure 3A) and greatest increase in plasma IGHV4 peptide usage from baseline (Figure 4). We used the LAX algorithm (18) to rank peptides in which the mass spectra matched VH/VL sequences (Supplemental Table 6) according to score and weight (defined in Methods). The peptide with the highest score belonged to the IGHV4 subgroup (Table 1 and Supplemental Table 6) and constituted a large fragment of CDR3 (20 out of 57 AA) (Figure 5).

### IGHV4 is the most frequent gene subgroup of Pfs25-specific single B cells.

**Figure 6:**
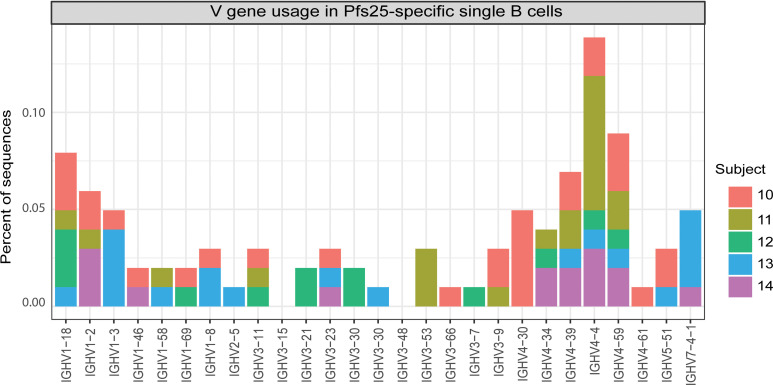
IGHV gene usage in Pfs25-specific single B cells collected from subjects after 4 doses of Pfs25-EPA/Alhydrogel. IGHV4 is the most frequently used IGHV subgroup for BcR of Pfs25-specific single B cells of vaccinees.

To further assess IGHV subgroup usage in response to Pfs25 vaccination, we isolated Pfs25-specific single B cells from subject 7 and from subjects 10–14 who received Pfs25-EPA/Alhydrogel in a second clinical trial in the same region of Mali (ClinicalTrials.gov ID NCT02334462) (Supplemental Table 7). We sequenced the V domain from B cells collected 14 days after dose 4 of the vaccine and obtained 495 sequences comprising 181 VH and 314 VL (287 κ and 27 λ) sequences (Supplemental Table 8). Among heavy chain sequences, the IGHV4 subgroup was the most frequently represented; in particular, the IGHV4-4 and IGHV4-59 genes were identified in samples from all subjects (Figure 6). Among light chain sequences, the IGKV4-1 gene was most frequently represented and seen in all 5 subjects, followed by the IGKV1D-39 gene seen in 4 of 5 subjects (Supplemental Figure 6).

### IGHV4 generates human mAbs that bind recombinant Pfs25.

**Figure 7:**
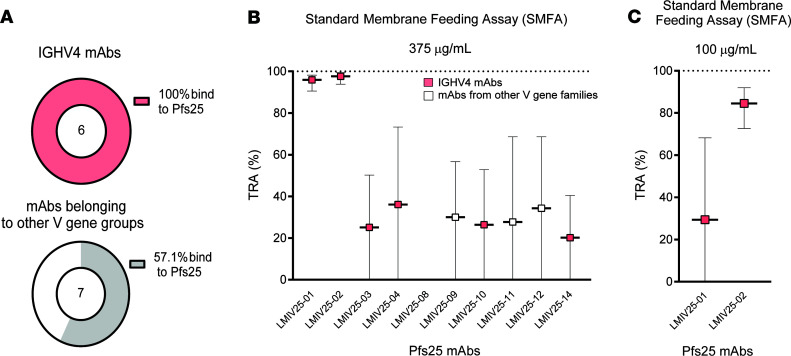
Human mAbs against Pfs25 using IGHV4 or other gene groups. (**A**) From the 13 mAbs tested, 10 bound to recombinant Pfs25 in ELISA –6 of 6 = in the IGHV4 group, and 4 of 7 in other groups. (**B**) Among the 10 binding mAbs, 2 (LMIV25-01 and -02) were highly functional at 375 ug/mL, both of which used IGHV4 sequences. (**C**) Only LMIV25-02 retained activity >80% at 100 ug/mL. Data are shown as mean ± SD.

Because the IGHV4 gene was the most frequent source of plasma Pfs25-IG peptides and of BcR for Pfs25-specific single B cells, we assessed whether IGHV4 usage was related to Pfs25 binding and functional activity. We generated recombinant human IgG1 mAbs using the VH and VL domain sequences obtained from subjects 10–14. Using 105 natural pairs of heavy and light chains obtained from the 96-well PCR plates, we selected 13 sequences for mAb expression based on evidence of clonal expansion. Of the 13 mAbs tested, 10 bound to recombinant Pfs25 by ELISA. Six of six IGHV4 mAbs bound to Pfs25, whereas four of seven using other IGHV subgroups bound to Pfs25 (Figure 7A). We then assessed the functionality and neutralizing profile of the 10 binding mAbs by evaluating their ability to reduce the parasite burden in *Anopheles* mosquitoes infected with *P*. *falciparum* NF54 strain (%TRA). Two of ten mAbs showed high functional activity when tested at 375 μg/mL (Figure 7B and Table 2). Both Abs belonged to the IGHV4 subgroup and were obtained from the same subject (subject 11) that yielded 2 nonfunctional mAbs (LMIV25-03 and -04). To confirm neutralizing activity, the 2 mAbs were tested at lower concentration, and LMIV25-02 was identified as a potent mAb with 84.4% TRA at 100 μg/mL (95% CI of the best estimate from 2 feeds: 72.6%–91.9%) (Figure 7C and Supplemental Table 9). Interestingly, LMIV25-02 presented evidence of affinity maturation when compared with the germline sequence (Supplemental Figure 7).

## Discussion

Here, we defined the plasma Ab response to a malaria vaccine at the molecular level using an innovative approach and multiple data sets. Specifically, we identified peptide sequences unique to antigen-specific plasma Abs produced by Malian adults after 4 doses of Pfs25-EPA/Alhydrogel. By matching plasma IG peptides to BcR sequences, we showed that V gene usage can be inferred for plasma Ab repertoire in response to a vaccine. We report for the first time to our knowledge plasma IG peptide sequences that match exactly to CDR3 of an antigen-specific single B cell from the same individual.

Initially, we purified Pfs25-specific Ig from the plasma of vaccinees and isolated F(ab′)_2_ fragments from these Abs. By this approach, Pfs25 IgG titers can be followed to demonstrate the success of the purification; other approaches have been used to obtain antigen-specific Ab peptides, for example, digesting the Abs into F(ab′)_2_ and Fc fragments and then affinity purifying the F(ab′)_2_ (16). We then performed proteomic analyses (liquid chromatography-tandem mass spectrometry [LC-MS/MS]) and matched Pfs25-F(ab′)_2_ peptide mass spectra to sequences from 3 different databases: (a) the publicly available IMGT data set containing germline V regions, (b) IGH CDR3 fragments of vaccine trial subjects amplified from total PBMCs 14 days after dose 4, and (c) Pfs25-specific single BcR V domains 14 days after dose 4. We have extended the approach to characterize serum Ab responses to vaccines (19) by alignment of peptides to antigen-specific single BCR, and this can also be used to analyze circulating Abs involved in autoimmune or infectious diseases.

We evaluated IGHV subgroup usage first in the IGH CDR3 data set and found no differences in V gene usage as a function of vaccine antigen (Pfs25 or comparators) or time point (day 0 or dose 4). This may be attributed in part to the time point when PMBCs were collected in this study (14 days after dose 4); the plasmablast peak occurs approximately 7 days after vaccination. In addition, our approach to obtain the IGH CDR3 data set involved sequencing of total PBMCs; therefore, the repertoire of IgM. B cells can be abundant and contribute to noise (20, 21) and the results might not represent the features of the antigen-specific IgG response.

We were interested to determine whether vaccination changes the profile of Pfs25-specific IG in plasma, which mediates vaccine activity against parasites within the mosquito blood meal (5). Alignment of F(ab′)_2_ peptides from Pfs25-specific plasma IG to IGH CDR3 sequences from Pfs25 vaccinees showed an increased frequency of IGHV4 usage compared with baseline (Figure 4). Corroborating these findings, IGHV4 usage was higher than other VH sequences among Pfs25-specific single memory B cells of vaccinees, and 2 Pfs25-IG mass spectra matched a CDR3 peptide that shared identity to an IGHV4 mRNA sequence expressed by a Pfs25-specific B cell from the same donor. This establishes our methodology as sufficiently sensitive to identify the same CDR3 concomitantly in plasma and in single B cells of the same donor, overcoming the very low probability expected in light of high CDR3 mutation rates (22).

All IGHV4 Pfs25 mAbs expressed from sequences of Pfs25-specific single B cells, including the only mAb with high neutralizing activity, bound to recombinant Pfs25. The association between IG V gene usage and vaccine or mAb efficacy is an emerging field (23), and further experimental studies are needed to substantiate the hypothesis that usage of a specific gene or subgroup may determine an effective Ab response against a given pathogen. Studies published earlier this year provide new information in this area. In one study of 2 subjects that received yellow fever vaccine, antigen-specific mAbs showed preferential usage of the IGHV3-72 gene (24). mAbs using the IGHV3-72 gene comprised most of the repertoire of memory B cells collected at 6 different time points, and comprised approximately 50% of the entire repertoire at early time points after vaccination (24). Another study showed the pattern of V gene usage was different among BcR IgG mAbs isolated from American and Malawian women who did or did not transmit HIV to their infants (25). For HIV, studies show that the BcR IG repertoire of the naive population can contain sequences that bind to HIV-1 immunogens, suggesting that BcR sequences shared between naive B cells and B cells elicited in response to HIV-1 can benefit vaccine design; it may be more likely that such a naive B cell will respond and generate serum Ab during vaccination (26, 27).

For malaria vaccines, the importance of IG V gene usage has not yet been established. Until now, no other study to our knowledge has evaluated the repertoire of IG V gene usage in single B cells following malaria vaccination in multiple subjects. In addition, the relationship between IG V gene usage and functional activity has only previously been explored by studying BcR from B cells of malaria-infected or -immunized subjects (28). In a previous report, McLeod and colleagues generated Pfs25 mAbs based on sequences of plasmablasts collected from a single malaria-naive volunteer 7 days after vaccination with plant-expressed Pfs25 adjuvanted in Alhydrogel; IGHV3 was frequently used in plasmablast sequences from this subject and although 5 of 12 IGHV3 mAbs had TRA higher than 80%, the 2 IGHV4 mAbs that bound to Pfs25 were also highly functional (88.3% and 83.5% of TRA, respectively) (15).

Results from different trials should be compared in light of the hypothesis that distinct vaccine formulations may promote unique features of diversification via somatic hypermutation, leading to distinct patterns of Ab repertoires, including differences in IG V gene usage (29, 30). Differences in IGHC gene usage in specific Abs may also be considered for isotypes and effector properties (17). African populations have remarkable IGHG allele polymorphism, demonstrated serologically by the γ marker allotype diversity (31), IGHG3 nucleotide sequence analysis (32) and, more recently, proteomic analysis of peptides of the IGHG3 genes and alleles (33, 34). Future studies should explore whether the serum Ab response to vaccines is, at the molecular level, affected by IGHG polymorphisms.

By analyzing Abs elicited in response to a malaria vaccine, our findings confirm the specificity of our approach for identifying antigen-specific serum peptides, using a combination of proteomics (35), bulk BcR sequencing, and single B cell sequencing. Notably, changes in IG V gene usage after vaccination were determined using antigen-specific but not bulk sequencing, highlighting the value of IgG proteomics to infer specific responses. To inform the design of a more efficient vaccine, peptide and VH/VL sequences revealed in this study can be further characterized and the epitope of the functional Pfs25 mAb LMIV25-02 can be identified. This information will guide strategies on how to improve the design of vaccines that neutralize malaria parasites during mosquito sexual stages.

## Methods

### Human immunization and samples collection.

A total of 41 Malian adults were vaccinated with 4 doses of 47 μg Pfs25-EPA in Alhydrogel on days 0, 56, 112, and 480 as part of 2 trials — NCT01867463 (8) and NCT02334462 — using similar methods, and subject follow-up schedule included blood sampling to generate sera and immune cells used in this study. Subjects were vaccinated with Euvax-B (hepatitis B) and Menactra (meningococcus) for the comparator group. The subjects enrolled in the clinical trials were healthy nonpregnant adults (71.6% male; 28.4% female) aged 18–59 years (mean 35.8 years) with lifelong exposure to intense seasonal *P. falciparum* infection.

Sera from 9 subjects were tested for transmission blocking activity (%TRA) by standard membrane feeding assay (SMFA) as previously described (7) and identified as TRA positive if their sera were able to reduce parasite burden in the mosquito midgut. Plasma (4 mL) were obtained from blood of individuals immunized with Pfs25-EPA at day 494 (14 days after dose 4) for IgG proteomics assays. PBMC (on average 5 million cells per tube) were obtained from blood collections at days 0 and 494 (14 days after dose 4) from 79 individuals from both Pfs25 vaccine and comparator groups. PBMCs for isolation of Pfs25-specific single B cells were obtained from a second clinical trial performed at the same site (Bancoumana, Mali) using the same dose of Pfs25-EPA/Alhydrogel.

### Expression of recombinant Pfs25.

Pfs25M is a *Pichia pastoris*-expressed recombinant Pfs25 with a molecular mass of 18,713 D in an oxidized state. It contains no heterologous amino- or carboxyl-terminal amino acids and was produced as described previously (36) with the exception that a cation-chromatography column replaced the metal-affinity chromatography column. EPA is an *E*. *coli–*expressed recombinant immunogenic carrier protein (a detoxified form of exotoxin A from *Pseudomonas aeruginosa*) with a molecular mass of 66,975 D (37). The Pfs25-EPA conjugate was produced by reaction between thiolated-Pfs25M and maleimide-activated EPA that was then purified by size-exclusion chromatography as previously described (38).

### Pfs25-specific Ab purification.

Pfs25 affinity columns were prepared to purify Pfs25-specific IG. Briefly, recombinant Pfs25 was chemically cross-linked to N-hydroxysuccinimide–activated Sepharose beads (GE Healthcare) following the manufacturer’s instructions. Pfs25-specific Abs (including all isotypes and subclasses) were affinity-purified by applying individual plasma samples to a Pfs25-specific packed resin column. Bound Abs were eluted from the column with a low-pH buffer (Invitrogen), neutralized with 2 mol/L Tris buffer (pH 9.0), and dialyzed in PBS (pH 7.4). Purified Ab concentration was measured by NanoDrop spectrophotometer (ND-2000; Thermo Fisher Scientific).

### ELISA.

Using standard ELISA, levels of anti-Pfs25 Abs were compared among antigen-purified Abs from plasma, original plasma, and plasma after depletion of Pfs25-specific Abs to establish an IgG purification control. Plates were coated with 100 μL recombinant Pfs25 antigen expressed in *P*. *pastoris*. Plates were incubated overnight at 4°C and blocked with 320 μL Tris-buffered saline containing 5% powdered skim milk for 2 hours at room temperature. Plates were then washed 4 times with Tween-TBS. Plasma samples (dilutions 1:500 and 1:5000) were added to antigen-coated wells in triplicate and incubated for 2 hours at room temperature. Plates were washed and incubated with 100 μL anti-human IgG (phosphatase-labeled goat anti-human heavy and light chains; Kirkegaard & Perry Laboratories) for 2 hours at room temperature, washed with Tween-TBS Wash Buffer, followed by 20 minutes of incubation in the dark at room temperature with the substrate (100 μL of 5 mg *p*-nitrophenyl phosphate per well; MilliporeSigma). Plates were read at absorbances of 405 and 650 nm using SoftMax Pro7 (Molecular Devices).

### Mass spectrometry for Pfs25 IgG peptide sequencing.

Pfs25 purified IgG Abs were digested with FabRICATOR, containing IdeS enzyme, to obtain F(ab′)_2_ fragments, then fractionated by 1-dimensional SDS-PAGE. The F(ab′)_2_ fraction was excised from the gel and reduced with 20 mM DTT for 20 minutes at 60°C, alkylated with 50 mM iodoacetamide for 20 minutes in the dark, digested with trypsin in a ratio of 1:50 (enzyme/total protein), and incubated overnight at 37°C. Peptides were extracted from the gel and desalted using C18 zip tips. Trypsin-digested peptide mixtures were loaded onto a reverse phase C-18 precolumn in line with an analytical column (Acclaim PepMap, 75 μm × 15 cm, 2 μm, 100A). Peptides were separated using a gradient of 5%–30% of solvent B (0.1% formic acid, acetonitrile) for 75 minutes, and then to 95% solvent B for an additional 50 minutes. Peptides were analyzed in data-dependent mode, and the top 20 precursors were fragmented using dissociation with a collision energy of 35. The mass window for precursor ion selection was 2 Da, and a minimum of 5000 counts were needed to trigger the MS/MS. MS1 was acquired in the Orbitrap at a resolution of 60,000 and MS2 at 7500 in the Orbitrap as well. Peptides from the Fc region were not analyzed in this study.

### RNA extraction from PBMC samples.

RNA was extracted from PBMC samples, using the RNeasy Blood & Tissue Kit (QIAGEN) after thawing and washing. Briefly, samples were thawed in a 37°C water bath for approximately 1 minute. Samples were then centrifuged at 350*g*, at 21°C –23°C, for 10 minutes. The supernatant was then aspirated, and cells were resuspended in 1 mL 37°C 1× RPMI (no additives). Samples were again centrifuged at 350*g*, at 21°C –23°C, for 5 minutes. The supernatant was aspirated again, and cells were resuspended in 240 μL 1× PBS. A total of 40 μL of this suspension were aliquoted and processed immediately. RNA was extracted following the manufacturer’s suggested methods for the RNeasy Mini Kit. RNA was eluted into approximately 30 μL RNase-free water and either used immediately for cDNA synthesis or stored at –80°C until cDNA synthesis could be performed.

### cDNA synthesis.

Isolated RNA was used to synthesize cDNA using the Invitrogen SuperScript III First-Strand Synthesis SuperMix for qRT-PCR kit. Briefly, 8 μL RNA was mixed with 10 μL 2× RT Reaction Mix and 2 μL RT Enzyme Mix, incubated at 25°C for 10 minutes, and then 50°C for 30 minutes. The reaction was terminated at 85°C for 5 minutes and then chilled on wet ice. Then, 1 μL (2 U) of *E*. *coli* RNase H was added, and the mixture incubated at 37°C for 20 minutes. Resulting cDNA was then stored at –20°C until use.

### IGH CDR3 immunosequencing.

Sequencing of the IGH CDR3 was performed using the immunoSEQ Assay (Adaptive Biotechnologies). Extracted cDNA samples were amplified in a bias-controlled multiplex PCR, followed by high-throughput sequencing. Sequences were collapsed and filtered to identify and quantitate the absolute abundance of each unique IGH CDR3 for further analysis as previously described (39–41).

### IGH CDR3 sequence analysis.

Sequences resulting from the immunoSEQ Assay were filtered to remove sequences that were out of frame, did not have a V or J gene assignment, or were detected by only a single template. For construction of V gene usage histograms, for each IGHV gene or IGHV gene subgroup (17, 42), the proportion of all sequences that comprised that subgroup were calculated, where the proportion is the sum of all sequences of a particular V gene divided by the total number of sequences for that sample.

To assess IGHV usage in Pfs25-IG plasma peptides, we compared mass spectra from Pfs25-IG from 9 subjects to IGH CDR3 sequences at the baseline and after vaccination. The data were presented as violin plot and heatmap. Proportions of V Gene Segment (VGS) use were determined by dividing the total number of reads assigned to a specific IGHV in a subject by the total number of reads seen by the subject at a common time point. If a VGS was not seen in any subjects at either time point, it is not shown in the data; if a VGS is seen in at least 1 subject at 1 time point but not the other, it is included in each time point but with a value of 0 in the appropriate locations. To generate the heatmap, the proportions of VGS use at day 0 (baseline) were subtracted from those calculated from dose 4 to generate the difference in proportions demonstrated in the figure. Heatmap was generated using the R software for statistical significance with heatmap2 and ggplot packages, and the violin plot was generated using GraphPad Prism 8.

### Pfs25 B cell tetramer construction.

Tetramer for selection and sorting of Pfs25-specific B cells was produced as previously described (43, 44). Recombinant Pfs25 protein expressed in *Pichia pastoris* was chemically biotinylated and tetramerized with streptavidin with known molarity and previously labeled with phycoerythrin (PE) (Prozyme). Decoy tetramer to exclude non-Pfs25-specific B cells was produced by conjugating Streptavidin-PE to DL594 using a DL594 Protein Labeling Kit (Thermo Fisher Scientific).

### Enrichment of Pfs25-specific cells in PBMC.

PBMC from subjects 7, 10, 11, 12, 13, and 14 were thawed in a 37°C water bath, resuspended in complete RPMI, and then, later, PBS. Pfs25-PE tetramer at 1 μM was added to the PBMCs and incubated at 4°C protected from the light for 20 minutes. Cells were washed with PBS containing 10% FBS and incubated with anti-PE magnetic beads (Miltenyi Biotec) for 25 minutes. Four mL PBS were added. The suspension was then passed over magnetized LS columns for elution of Pfs25-PE–specific cells.

### Flow cytometry and sorting of Pfs25-specific B cells.

After the enrichment with Pfs25 and decoy tetramers, human cells were stained with CD3- (UCHT1), CD14- (M5E2), CD56- (HCD56) Alexa Fluor 700–, CD19 APC-CY7– (HIB19), and CD20 PE-CY7–conjugated (2H7) Abs purchased from BioLegend.

A gating strategy was performed first for doublet discrimination, then singlet cells were selected for exclusion of non–B cells using CD3, CD14, and CD56 markers. Lymphocytes were gated for CD19^+^CD20^+^. Pfs25-specific B cells were gated using PE and excluding non-Pfs25 B cells in CF594, the fluorochrome used in the decoy BSA tetramer. Analysis was performed in FACSAria II instrument (BD Biosciences) with blue, red, and violet lasers. Pfs25-specific B cells were analyzed according to the fluorescence staining profile previously described and sorted directly into a 96-well plate using FACS with a nozzle of 100 μM. Plates were then centrifuged at 1278*g* for 30 seconds and placed into a –80°C freezer.

### IG VH and VL sequencing of Pfs25-specific single B cells.

Amplification of IG heavy (VH) and light (VL) domains from single sorted cells was performed by iRepertoire Inc. Briefly, RT-PCR1 was performed with nested, multiplex primers covering heavy, κ, and λ loci, and including partial Illumina adaptors. A total of 500 bp fragments corresponding to the VH and to the VL domains were amplified from each single B cell.

### Expression and purification of recombinant mAbs.

VH and VL sequences of Pfs25-specific single B cells (CD19^+^CD20^+^) were selected for expression based on evidence of activation. If multiple cells contained identical CDR3 sequences, it was considered to have been attributed to clonal expansion, and thus evidence of activation. Naturally paired VH/VL sequences were expressed in an IgG1 backbone by LakePharma Inc. IgG1 mAbs were expressed using 0.1 L transient production in HEK293 cells (Thermo Fisher Scientific) and purified using protein A affinity chromatography. Purified proteins were submitted to sterile filtering using a 0.2 μm sterile filter and characterized for more than 90% purity by capillary electrophoresis-SDS, concentration, and endotoxin.

### ELISA for Pfs25 mAbs.

mAb responses against recombinant Pfs25 were measured using ELISA. Immulon 4HBX plates were coated with 1 μg per well of recombinant Pfs25. Plates were incubated overnight at 4°C and blocked with 320 μL buffer containing 5% skim milk powder in Tris-buffered saline for 2 hours at room temperature. Plates were washed with Tween-TBS. Samples (dilution 1:500) were added to Pfs25-coated wells, in triplicate, and incubated for 2 hours at room temperature. Plates were washed, then 100 μL alkaline phosphatase-labeled goat anti-human IgG were added. Plates were incubated for 2 hours at room temperature and washed. A colorimetric substrate, *p*-nitrophenyl phosphate (MilliporeSigma) was added, and plates were read at absorbances of 450 and 550 nm on a multi-well reader (Molecular Devices).

### SMFA.

SMFA was performed to assess the ability of mAbs to block the development of *P*. *falciparum* strain NF54 oocysts in the mosquito midgut. For this, stage V gametocytes from a mature gametocyte culture were mixed with normal human serum and normal red blood cells to make a feeding mixture with 0.15%–0.2% stage V gametocytemia. SMFAs were conducted in the presence of active human complement. Purified mAbs were added to these at the specified concentrations and then fed to 3- to 6-day-old starved female *Anopheles stephensi* (SDA 500, NIAID) via a parafilm membrane. The mosquitoes were maintained for 8 days and then dissected to count the number of oocysts per midgut in 20 mosquitoes. Percent reduction in mean infection intensity (%TRA) was calculated relative to the respective control IgG tested in the same assay.

### Bioinformatic analysis of LC-MS/MS data.

Pfs25-specific plasma IG F(ab′)_2_ tryptic peptides (proteome data sets of 9 subjects) were analyzed by mass spectrometry (LTQ Orbitrap Velos), and acquired spectra were analyzed using PEAKS Studio 8.5 (Bioinformatics Solutions Inc.) using a database composed of the following: (a) from IMGT(http://www.imgt.org [ref. 42], containing the V-region translation of *Homo sapiens* germline genes and alleles: 303 IGHV [average sequence size 93.53 AA], 83 IGLV [average sequence size 95.90 AA], and 89 IGKV sequences [average sequence size 94.11 AA]), ref. 17; (b) an in-house IGH CDR3 data set generated by translation of 3.15 million IGH CDR3 sequences amplified from the PBMCs of 79 subjects (vaccinees and comparators, mean of 39,966 sequences per subject); and (c) a data set containing 22 VH and VL sequences of Pfs25-specific single B cells (single-cell data set) from subject 7 were searched against the Pfs25-specific plasma IG F(ab′)_2_ peptides from the same donor. Nucleotide sequences were translated to amino acids using the R function “DNAtoBestPeptide()” in the R package DuffyTools. The cRAP (common Repository of Adventitious Proteins) database was used in all databases to exclude possible contaminants. The V modifications selected were carbamidomethyl (C), deamidation (NQ), and oxidation (M), and 2 missed cleavage sites were allowed. The false discovery rate (FDR) for peptides was set to 1% by applying the target-decoy strategy.

The PEAKS software matches MS/MS spectra to peptide sequences in each database by employing de novo peptide sequencing to improve speed and accuracy of the database search. In addition, PEAKS, as with other proteomics software, uses the target-decoy strategy to compare correct (target) peptide-spectra matches to incorrect (decoy) peptide-spectra matches and generates a FDR (45).

### LAX algorithm.

VH/VL sequences from Pfs25-specific single B cells (single-cell data set) were searched in the Pfs25-specific plasma IG F(ab′)_2_ peptides (proteome data set) from the same donor using a novel local peptide alignment algorithm (LAX) (46) that uses the R “pairwiseAlignment()” function from the ‘Biostrings’ package (47, 48). By using a modified version of the PAM70 substitution matrix, altered to give nonnegative penalties to the most common spectra misassignments (Ile vs. Leu, Glu vs. Gln, and Asp vs. Asn), LAX can tolerate mismatches and gaps in the F(ab′)_2_ fragments of MS/MS-generated peptides to find the best scoring assignment to 1 or more Ig protein sequences. This facilitates finding the best IG identity for peptides without the need for knowing a priori the complete repertoire of IG sequences in a subject.

Each peptide is scored by LAX against protein sequences from the database containing single BcR IG V-(D)-J sequences from subject 7, plus the cRAP database, to identify 1 or more highest scoring protein matches for each peptide. The scores reported by the LAX algorithm include: weight for each peptide, based on the number of equally high scoring protein hits that LAX found, where 1.0 signifies a unique assignment to 1 and only 1 protein, and lower weights for peptides matching 2 or more proteins; percent match for each peptide, a value that quantifies the percentage of amino acids in the peptide that are perfect matches to the assigned protein sequence, where again 1.0 signifies a perfect match, and lower percent match scores for peptides with increasing numbers of mismatches, insertions, or deletions. In addition, LAX reports a ScorePerAA that normalizes the final PAM70 score by peptide length, to adjust for different length peptides. Peptides with a ScorePerAA of less than 4.3 were discarded, for being too dissimilar to any database protein.

### Statistics.

Paired *t* test (2 tailed) was used to compare data from different experimental groups. *P* values of less than 0.05 were considered significant.

### Study approval.

NCT01867463 and NCT02334462 (ClinicalTrials.gov) clinical trials were approved by the ethics review boards from the Faculté de Médecine, de Pharmacie et d’OdontoStomatologie (FMPOS) Bamako, Mali; the US National Institute of Allergy and Infectious Diseases (NIH, Bethesda, Maryland, USA); and the Mali National Regulatory Authority (Bamako, Mali). The safety and immunogenicity results of phase I from the NCT01867463 clinical trial are published elsewhere (8).

## Author contributions

CHC, MF, and PED conceived the study. CHC, PGH, MF, YW, MV, and PED designed the experiments. CHC, PGH, JYD, MSS, JL, NJM, and KM performed experiments. CHC, STN, PGH, RM, and JDG analyzed the data. PED, MF, JR, MV, CS, DLN, YW, CRK, GP, SWM, CL, IS, MLF, and JT supervised the experiments or analyses. All authors interpreted the data. CHC and PED wrote the manuscript with input from all authors.

## Supplementary Material

supplemental data

supplemental Table 1

supplemental Table 2

supplemental Table 3

supplemental Table 4

supplemental Table 5

supplemental Table 6

supplemental Table 7

supplemental Table 8

## Figures and Tables

**Table 2:**
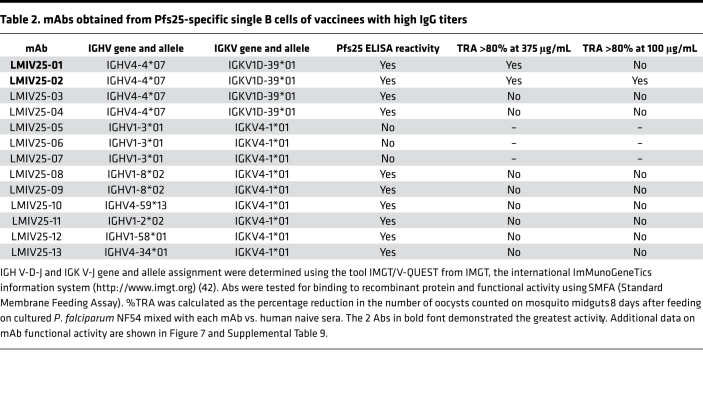
mAbs obtained from Pfs25-specific single B cells of vaccinees with high IgG titers

**Table 1:**
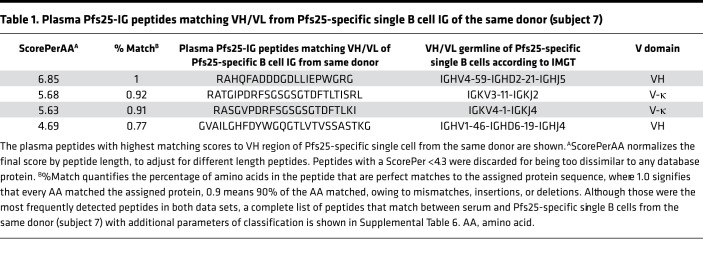
Plasma Pfs25-IG peptides matching VH/VL from Pfs25-specific single B cell IG of the same donor (subject 7)

## Version 1 10/13/2020

In-Press Preview

## Version 2 11/19/2020

Electronic publication

## References

[1] Rabinovich RN (2017). malERA: an updated research agenda for malaria elimination and eradication. PLoS Med.

[2] Coelho CH, Doritchamou JYA, Zaidi I, Duffy PE. Advances in malaria vaccine development: report from the 2017 malaria vaccine symposium. *NPJ Vaccines*. 2017;2:34 10.1038/s41541-017-0035-3 PMC5709382 29522056

[3] Long CA, Zavala F (2016). Malaria vaccines and human immune responses. Curr Opin Microbiol.

[4] Gwadz RW, Carter R, Green I (1979). Gamete vaccines and transmission-blocking immunity in malaria. Bull World Health Organ.

[5] Coelho CH, Rappuoli R, Hotez PJ, Duffy PE (2019). Transmission-blocking vaccines for malaria: time to talk about vaccine introduction. Trends Parasitol.

[6] Coelho CH (2019). Chronic helminth infection does not impair immune response to malaria transmission blocking vaccine Pfs230D1-EPA/Alhydrogel® in mice. Vaccine.

[7] Talaat KR (2016). Safety and immunogenicity of Pfs25-EPA/Alhydrogel®, a transmission blocking vaccine against *Plasmodium falciparum*: an open label study in malaria naïve adults. PLoS One.

[8] Sagara I (2018). Safety and immunogenicity of Pfs25H-EPA/Alhydrogel, a transmission-blocking vaccine against *Plasmodium falciparum*: a randomised, double-blind, comparator-controlled, dose-escalation study in healthy Malian adults. Lancet Infect Dis.

[9] Kapoor N (2018). Malaria derived glycosylphosphatidylinositol anchor enhances anti -Pfs25 functional antibodies that block malaria transmission. Biochemistry.

[10] Eldering M (2017). Comparative assessment of An. gambiae and An. stephensi mosquitoes to determine transmission-reducing activity of antibodies against *P*. *falciparum* sexual stage antigens. Parasit Vectors.

[11] Scally SW (2017). Molecular definition of multiple sites of antibody inhibition of malaria transmission-blocking vaccine antigen Pfs25. Nat Commun.

[12] Galson JD (2015). BCR repertoire sequencing: different patterns of B-cell activation after two Meningococcal vaccines. Immunol Cell Biol.

[13] Galson JD (2015). Analysis of B cell repertoire dynamics following hepatitis B vaccination in humans, and enrichment of vaccine-specific antibody sequences. EBioMedicine.

[14] Steichen JM (2019). A generalized HIV vaccine design strategy for priming of broadly neutralizing antibody responses. Science.

[15] McLeod B (2019). Potent antibody lineage against malaria transmission elicited by human vaccination with Pfs25. Nat Commun.

[16] Lavinder JJ (2014). Identification and characterization of the constituent human serum antibodies elicited by vaccination. Proc Natl Acad Sci U S A.

[17] Lefranc MP, Lefranc G. *The Immunoglobulin FactsBook*. Academic Press; 2001.

[18] Gonzales Hurtado PA (2019). Proteomics pipeline for identifying variant proteins in *Plasmodium falciparum* parasites isolated from children presenting with malaria. J Proteome Res.

[19] Lee J (2016). Molecular-level analysis of the serum antibody repertoire in young adults before and after seasonal influenza vaccination. Nat Med.

[20] Chaudhary N, Wesemann DR (2018). Analyzing immunoglobulin repertoires. Front Immunol.

[21] Wu YC, Kipling D, Leong HS, Martin V, Ademokun AA, Dunn-Walters DK (2010). High-throughput immunoglobulin repertoire analysis distinguishes between human IgM memory and switched memory B-cell populations. Blood.

[22] Xu JL, Davis MM (2000). Diversity in the CDR3 region of V(H) is sufficient for most antibody specificities. Immunity.

[23] Kreer C, Gruell H, Mora T, Walczak AM, Klein F (2020). Exploiting B cell receptor analyses to inform on HIV-1 vaccination strategies. Vaccines (Basel).

[24] Wec AZ (2020). Longitudinal dynamics of the human B cell response to the yellow fever 17D vaccine. Proc Natl Acad Sci U S A.

[25] Martinez DR (2020). Maternal broadly neutralizing antibodies can select for neutralization-resistant, infant-transmitted/founder HIV variants. mBio.

[26] Havenar-Daughton C, Abbott RK, Schief WR, Crotty S (2018). When designing vaccines, consider the starting material: the human B cell repertoire. Curr Opin Immunol.

[27] Havenar-Daughton C (2018). The human naive B cell repertoire contains distinct subclasses for a germline-targeting HIV-1 vaccine immunogen. Sci Transl Med.

[28] Julien JP, Wardemann H (2019). Antibodies against *Plasmodium falciparum* malaria at the molecular level. Nat Rev Immunol.

[29] Galson JD, Trück J, Kelly DF, van der Most R (2016). Investigating the effect of AS03 adjuvant on the plasma cell repertoire following pH1N1 influenza vaccination. Sci Rep.

[30] Wrammert J (2008). Rapid cloning of high-affinity human monoclonal antibodies against influenza virus. Nature.

[31] Lefranc MP, Lefranc G (2012). Human Gm, Km, and Am allotypes and their molecular characterization: a remarkable demonstration of polymorphism. Methods Mol Biol.

[32] Dard P, Lefranc MP, Osipova L, Sanchez-Mazas A (2001). DNA sequence variability of IGHG3 alleles associated to the main G3m haplotypes in human populations. Eur J Hum Genet.

[33] Dechavanne C (2012). Mass spectrometry detection of G3m and IGHG3 alleles and follow-up of differential mother and neonate IgG3. PLoS One.

[34] Dambrun M (2017). Human immunoglobin heavy gamma chain polymorphisms: molecular confirmation of proteomic assessment. Mol Cell Proteomics.

[35] Wine Y, Horton AP, Ippolito GC, Georgiou G (2015). Serology in the 21st century: the molecular-level analysis of the serum antibody repertoire. Curr Opin Immunol.

[36] Tsai CW, Duggan PF, Shimp RL, Miller LH, Narum DL (2006). Overproduction of *Pichia pastoris* or *Plasmodium falciparum* protein disulfide isomerase affects expression, folding and O-linked glycosylation of a malaria vaccine candidate expressed in *P*. *pastoris*. J Biotechnol.

[37] Burkhardt M (2019). Assessment of the impact of manufacturing changes on the physicochemical properties of the recombinant vaccine carrier exoprotein A. Vaccine.

[38] Shimp RL (2013). Development of a Pfs25-EPA malaria transmission blocking vaccine as a chemically conjugated nanoparticle. Vaccine.

[39] DeWitt WS (2016). A public database of memory and naive B-cell receptor sequences. PLoS One.

[40] Wu D (2014). Detection of minimal residual disease in B lymphoblastic leukemia by high-throughput sequencing of IGH. Clin Cancer Res.

[41] Carlson CS (2013). Using synthetic templates to design an unbiased multiplex PCR assay. Nat Commun.

[42] Lefranc MP (2014). Immunoglobulin and T cell receptor genes: IMGT(®) and the birth and rise of immunoinformatics. Front Immunol.

[43] Krishnamurty AT (2016). Somatically hypermutated plasmodium-specific IgM(+) memory B cells are rapid, plastic, early responders upon malaria rechallenge. Immunity.

[44] Taylor JJ (2012). Deletion and anergy of polyclonal B cells specific for ubiquitous membrane-bound self-antigen. J Exp Med.

[45] Zhang J (2012). PEAKS DB: de novo sequencing assisted database search for sensitive and accurate peptide identification. Mol Cell Proteomics.

[46] Gonzales Hurtado PA (2019). Proteomics pipeline for identifying variant proteins in *Plasmodium falciparum* parasites isolated from children presenting with malaria. J Proteome Res.

[47] Gentleman RC (2004). Bioconductor: open software development for computational biology and bioinformatics. Genome Biol.

[48] R Core Team. R: a language and environment for statistical computing. R Foundation for Statistical Computing, Vienna, Austria. http://www.R-project.org/ Accessed October 13, 2020

