# Supplementary material for: Antimalarial antibody repertoire defined by plasma IG proteomics and single B cell IG sequencing
Source: JCI Insight. 2020 Nov 19;5(22):e143471. doi: 10.1172/jci.insight.143471 (PMC7710313; doi:10.1172/jci.insight.143471)
Supplement: supplemental data [file jciinsight-5-143471-s173.pdf]

SUPPLEMENTAL DATA

FIGURES

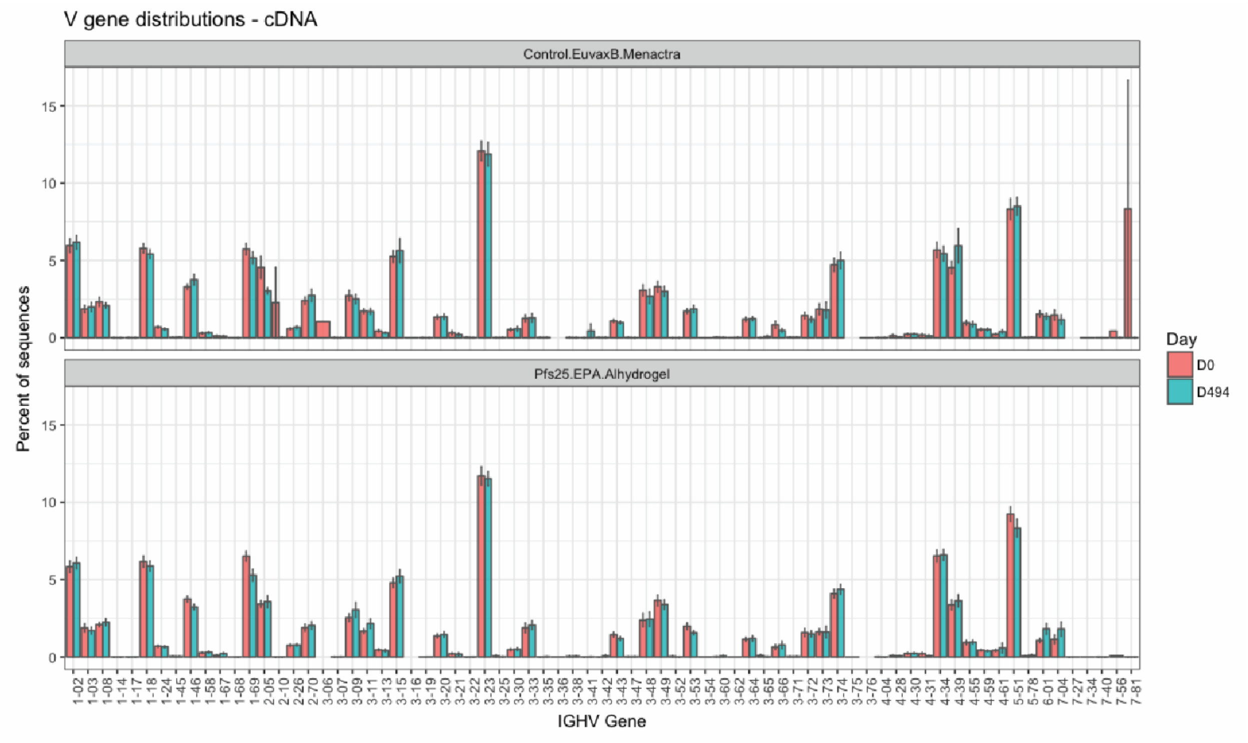

Supplemental Figure 1 – V gene usage among vaccinees (Pfs25-EPA/Alhydrogel®) and comparators (Euvax-B and Menactra®) at days 0 (baseline) and 494 (14 days after the 4th dose).

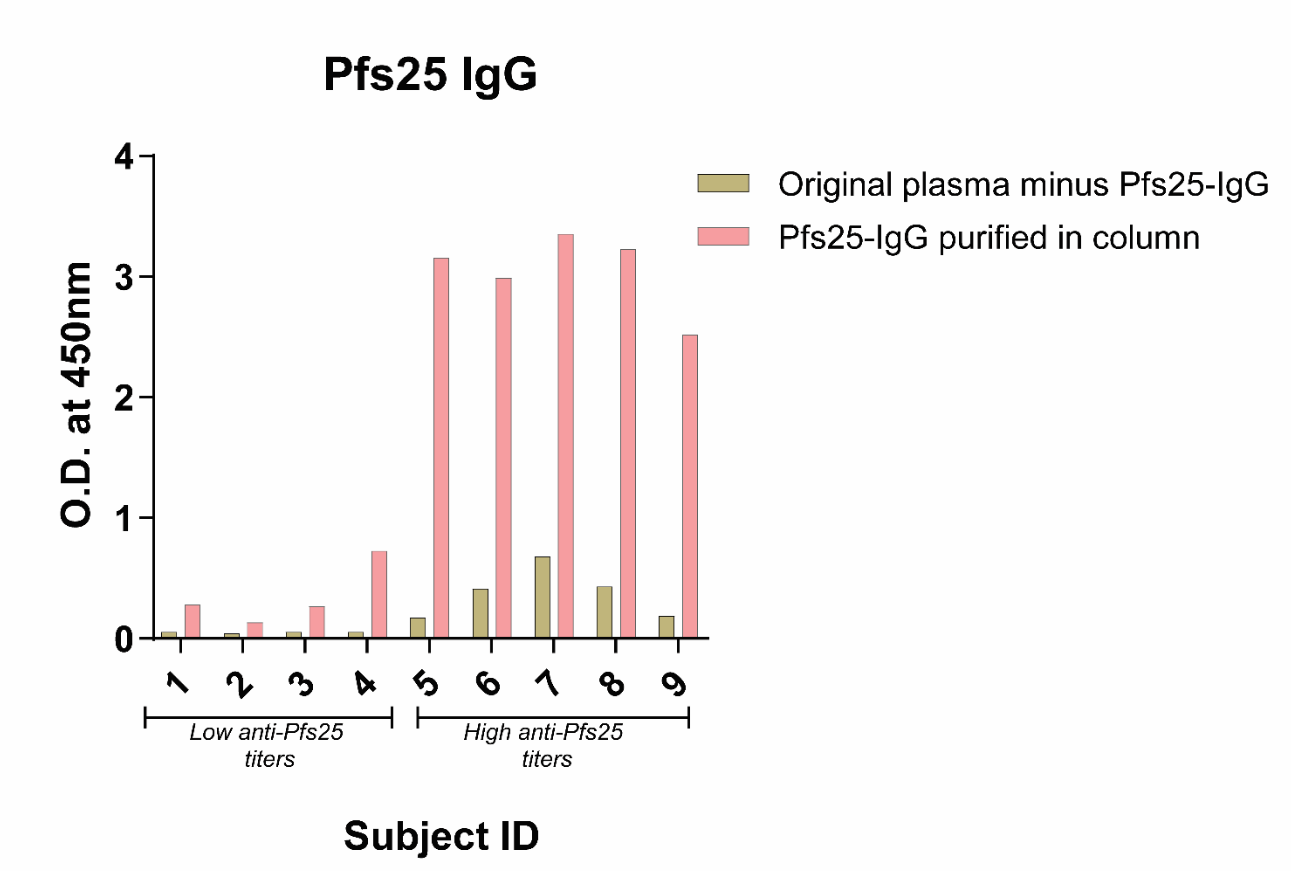

Supplemental Figure 2 - Anti-Pfs25 IgG levels in Pfs25-depleted plasma and in corresponding Pfs25-IG antibody samples. Information about each subject's original antibody titers is provided in Table 1.

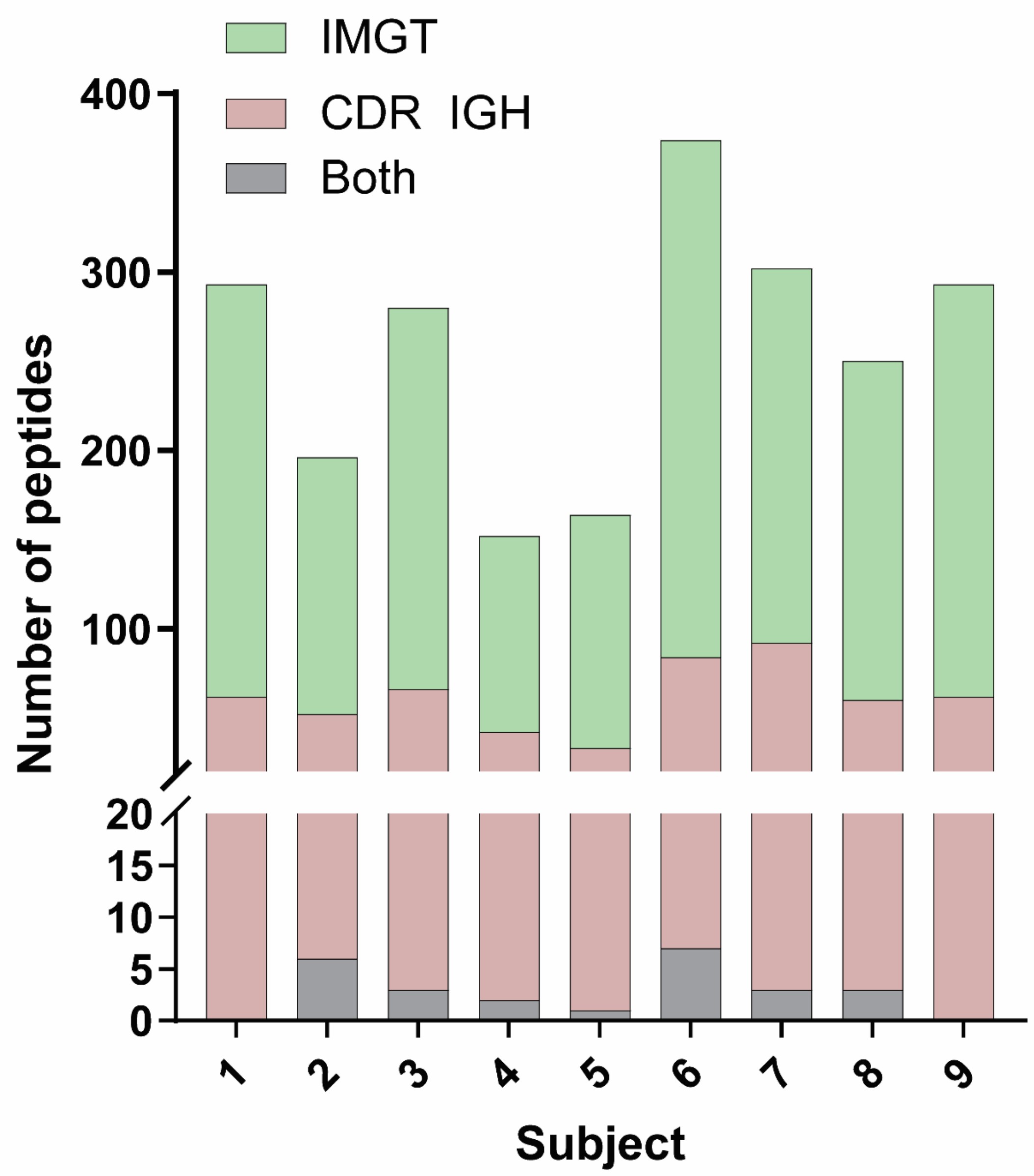

Supplemental Figure 3 - Peptides matching to both the IMGT® database and IGH CDR3 dataset. Peptide counts were obtained after removing duplicates and posttranslational (PTM) notations and the full list of peptide-spectrum matches is shown in Supplemental Table 2. The number of matches was higher in the IMGT® database (which includes sequences of the complete germline variable region) than in the IGH CDR3 dataset (which includes sequences of the shorter CDR3 fragments).

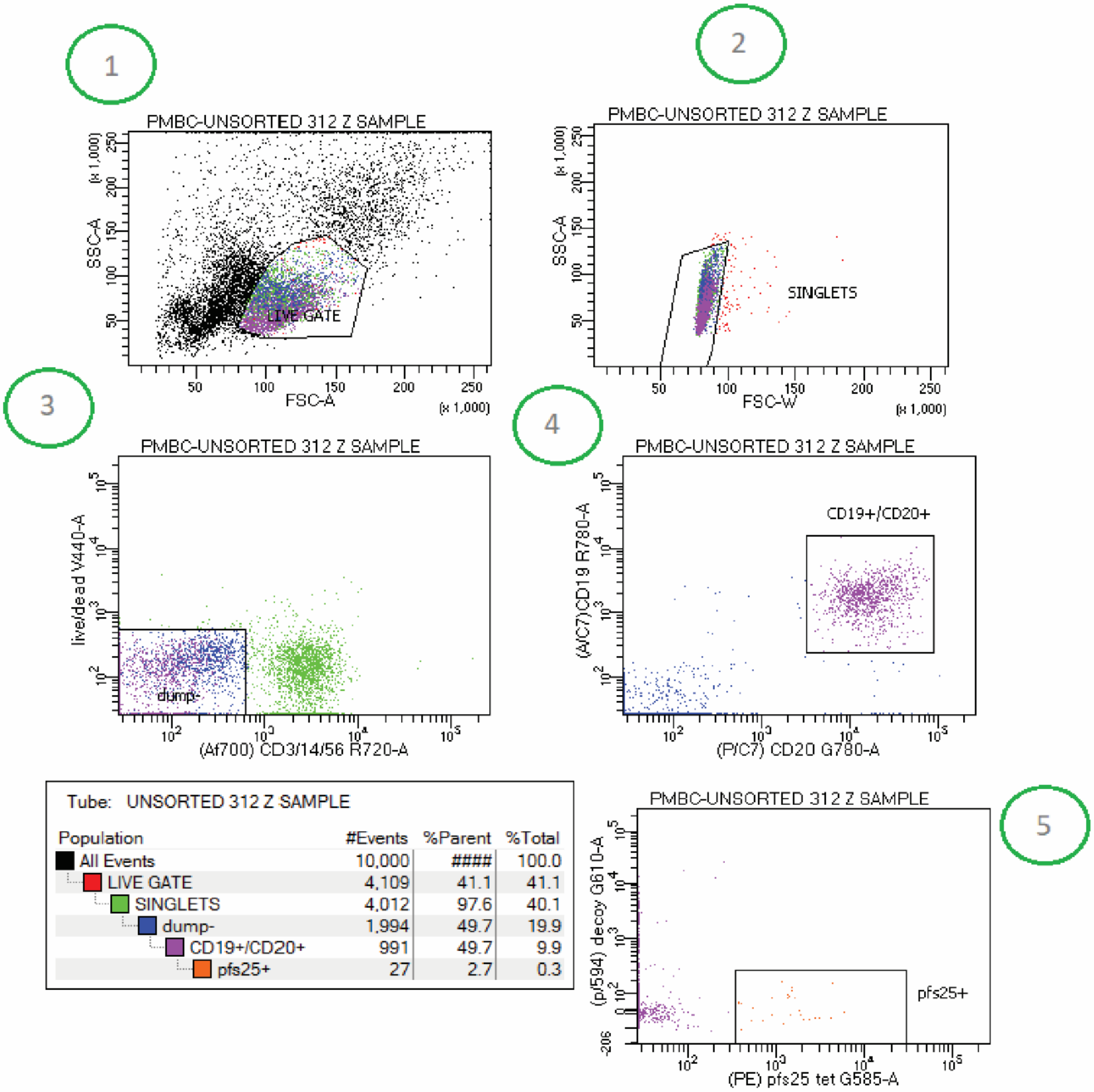

Population	#Events	%Parent	%Total
All Events	10,000	####	100.0
LIVE GATE	4,109	41.1	41.1
SINGLETS	4,012	97.6	40.1
dump-	1,994	49.7	19.9
CD19+/CD20+	991	49.7	9.9
pfs25+	27	2.7	0.3

Supplemental Figure 4 – Flow cytometry gating strategy to sort individual Pfs25-specific B cells for further sequencing of VH/VL domains. Numbers in the circles show sequence for gating of Pfs25-specific single B cells. The bottom right panel identifies the antigen-specific single B cells.

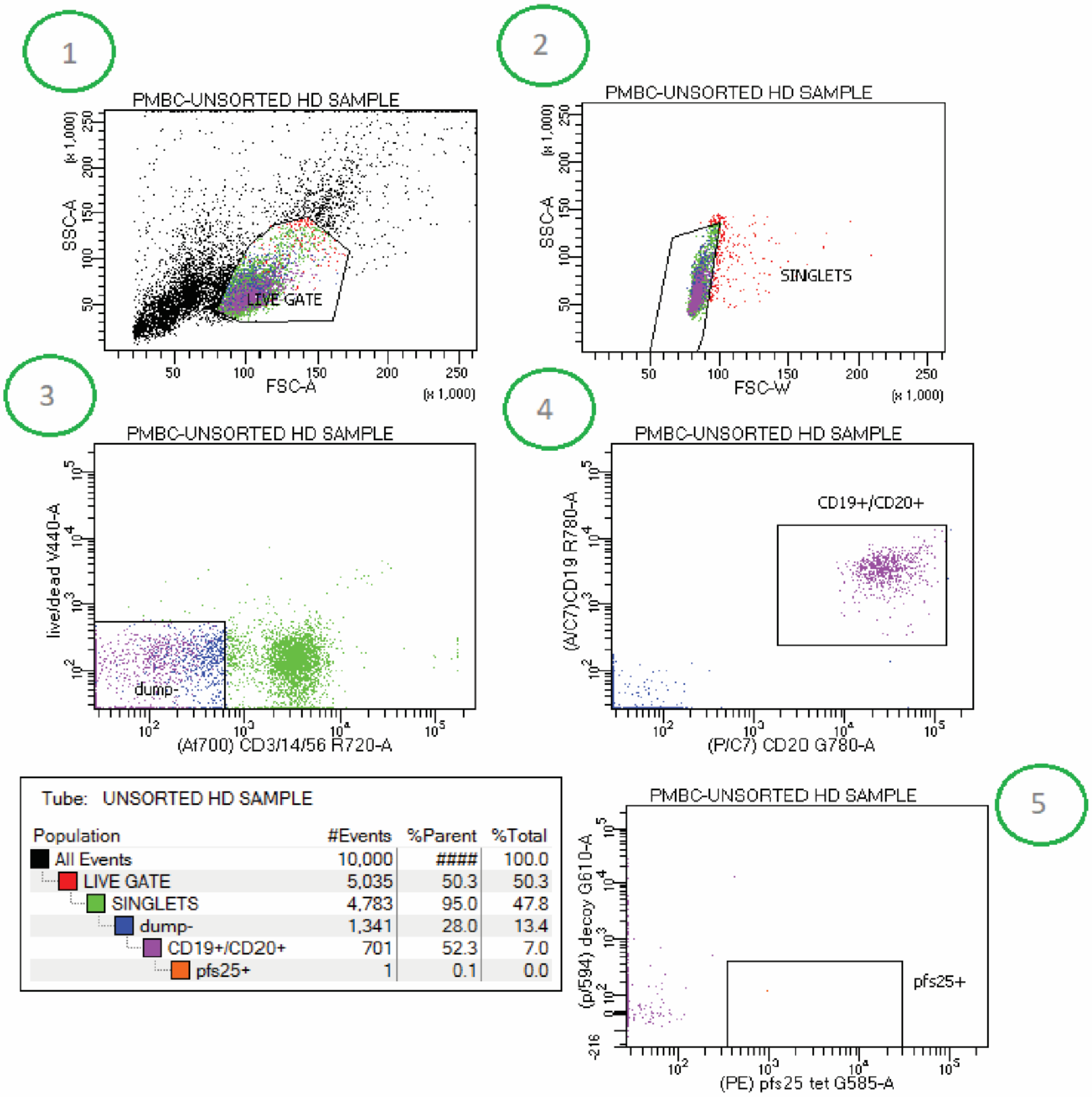

Supplemental Figure 5 – Flow cytometry gating strategy using PBMCs of healthy donors to confirm non-binding to Pfs25 and tetramer specificity. Numbers in the circles show sequence for gating of Pfs25-specific single B cells. The bottom right panel identifies the antigen-specific single B cells.

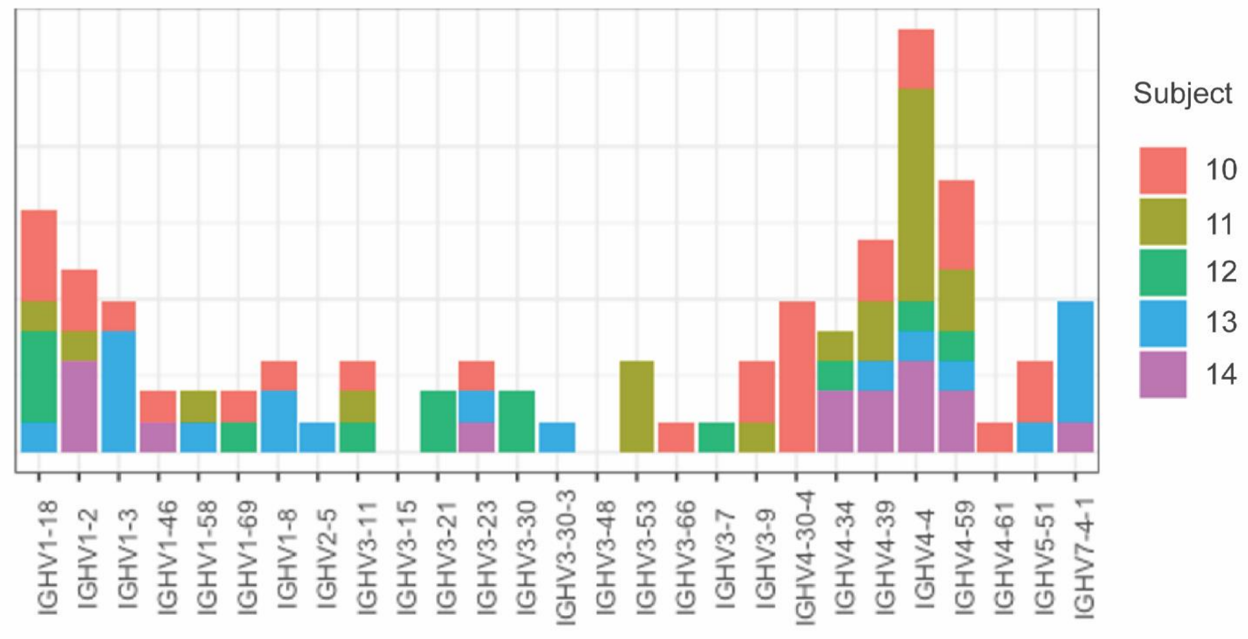

Supplemental Figure 6 – V gene usage in light chain sequences (kappa and lambda) of Pfs25-specific single B cells of five subjects. Each color represents one subject. TRA (functional activity) for each subject is shown at **Supplemental Table 4.**

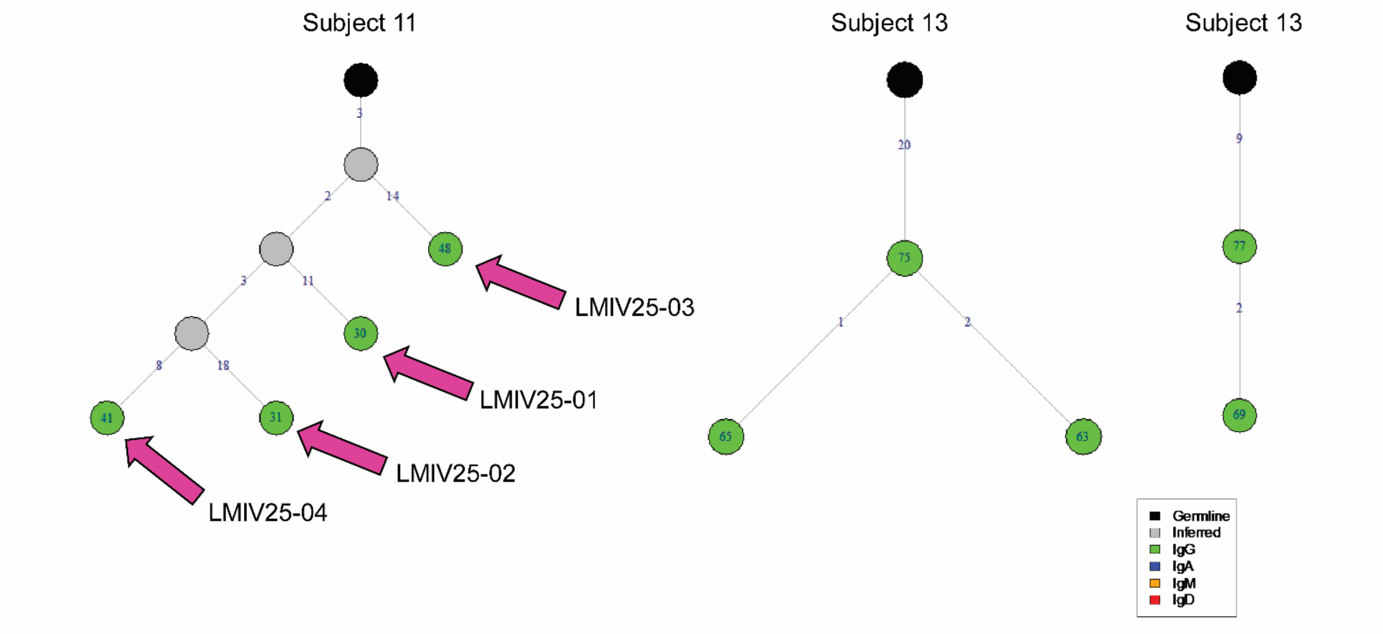

Supplemental Figure 7 – VH sequences of Pfs25-specific single B cells rooted to the germline. Sequences were defined as being part of the same clonal expansion if they shared the same V and J genes, and they were >90% similar in their CDR3 sequence. Black circles represent germline sequences, gray circles inferred sequences, and green circles the ID number of the VH sequences identified. Blue numbers in the lines represent number of somatic hypermutations compared to the germline. Maximum parsimony tree was drawn using the R package *alakazam*. mAb sequences are denoted by the pink arrows.

SUPPLEMENTAL DATA

TABLES

Supplemental Table 1 – t test analyses from Figure 1.

Supplemental Table 2 – PEAKS Studio 8.5 result files of plasma Pfs25-IG peptides from plasma of Pfs25-EPA/Alhydrogel® vaccinees matched to IGH sequences from the IGH CDR3 dataset or IMGT® database. Tabs are specific to each subject, wherein each tab contains an Accession ID, tryptic peptide sequence with modifications, PEAKS score (-10logP), peptide mass, peptide AA length, charge, number of spectra, and post-translational modifications (PTMs) detected. Regarding the IMGT® translated germline reference sequences, accession IDs containing "F" correspond to "Functional", "ORF" corresponds to "Open reading frame" and "P" corresponds to "Pseudogene" (according to IMGT® functionality). In the Peptide column, periods represent tryptic cleavage sites and in parentheses charge and extra mass are shown corresponding to a PTM.

Supplemental Table 3 – Plasma Pfs25-IG peptides from Pfs25-EPA/Alhydrogel® vaccinees that mapped exclusively to IGH CDR3 sequences from Pfs25 vaccinees at baseline (day 0) and dose 4 (day 494, 14 days after the 4th dose).

Supplemental Table 4 – Raw data used to generate the violin plot at Figure 4a. Proportion of all Pfs25-IG plasma peptides that can be assigned to an IGHV subgroup in the IGH CDR3 dataset.

Supplemental Table 5 – Raw data used to generate the heatmaps at Figure 4b. Difference in the proportion of plasma Pfs25-IG peptides that can be attributed to an IGHV subgroup after dose 4 versus day 0 (baseline).

Supplemental Table 6 – Pfs25-specific plasma IG F(ab')2 peptides from subject 7 matching VH and VL sequences of Pfs25-specific single B cells from the same donor. Sequence highlighted in blue was mapped in a large portion of CDR3.

Supplemental Table 7 – Titers and functional activity (TRA, Transmission-Reducing Activity) for donors of the PBMC used for isolation of Pfs25-specific single B cell.

Supplemental Table 8 – VH and VL sequences obtained from sequencing of Pfs25-specific single B-cells of subjects 10 to 14.

Supplemental Table 9 – Standard Membrane Feeding Assay data for the mAbs LMIV25-01 and 02 at 100 ug/mL.
